# ICD-11 Prolonged Grief Disorder, Physical Health, and Somatic Problems: A Systematic Review

**DOI:** 10.32872/cpe.14351

**Published:** 2025-02-28

**Authors:** James Cunningham, Mark Shevlin, Catalina Cerda, Eoin McElroy

**Affiliations:** 1 School of Psychology, Ulster University, Coleraine, United Kingdom; Philipps-University of Marburg, Marburg, Germany

**Keywords:** prolonged-grief, bereavement, PGD, physical, somatic, somatization, illness

## Abstract

**Background:**

Since Prolonged Grief Disorder’s (PGD) inclusion as a mental health disorder in the ICD-11 in 2018, much of the peer-reviewed research has focused on its prevalence, assessment, and co-occurrence with other mental health disorders. There is also emerging research literature on the association between PGD and physical and somatic health outcomes. In light of this, the objective of this review was to identify and summarise the extant research on the association between PGD, and outcomes related to physical health and somatic complaints among bereaved individuals.

**Method:**

A systematic review utilized electronic databases (Web of Science, MEDLINE, Cochrane Library, PsycINFO) up to October 10, 2023. Included were cohort and cross-sectional studies since 2018 exploring links between ICD-11 PGD and physical/somatic health outcomes. Two researchers independently identified eligible studies meeting inclusion/exclusion criteria, employing quality assessment instruments to evaluate methodological rigor.

**Results:**

From the 418 articles that were initially screened, 18 met the inclusion criteria. The studies reported significant associations between PGD and physical health, somatic symptom distress, insomnia severity, blood pressure, bodily distress syndrome, chronic physical diseases, and poor- caregiver health profiles.

**Conclusion:**

Out of the 18 studies eligible for analysis, 13 (72%) established a significantly strong or moderate association between PGD and physical or somatic illness, highlighting the intricate nature of this connection. Further research is required to assess the breadth of physical and somatic health problems associated with PGD and to understand the psychological and biological mechanisms that underpin these observed relationships.

## Highlights

- Most studies found a strong to moderate link between PGD and physical/somatic illness.
- PGD impacts caregiver health, somatic distress, insomnia, and comorbid chronic diseases.
- Findings align with PTSD, with clinically relevant psychological and medical effects.

Physical health is defined by the Centers for Disease Control and Prevention as the condition of one’s body, with the ability to carry out daily activities without experiencing pain, discomfort, or limitation (Elgaddal et al., 2022). Somatic problems, on the other hand, are physical symptoms that are not caused by an identifiable medical condition (Kolappa et al., 2013). The association between mental health disorders and physical health or somatic symptoms has been consistently reported in the research literature. For example, depression has been identified as a risk factor for long-term physical conditions such as diabetes (Cosgrove et al., 2008; Gonzalez et al., 2008), cancer (Massetti et al., 2017; Massie, 2004), and cardiac disease (Berg et al., 2018; Chaddha et al., 2016; Dhar & Barton, 2016).

Studies conducted by Haug et al. (2004) and Carlehed et al. (2017) have also explored how depression relates to physical symptoms in large community samples, revealing a strong and significant relationship between depression and experiencing functional somatic symptoms. Moreover, Gili et al. (2010) reported a higher prevalence of depression among primary care patients with chronic somatic diseases compared to their physically healthy counterparts.

One disorder that has consistently been found to be associated with physical and somatic problems is post-traumatic stress disorder (PTSD). The ICD-11 outlines PTSD as a mental health condition that can emerge after experiencing a threatening or horrifying event or a sequence of such events (Barbano et al., 2019) and the associated allostatic load has been argued to cause physical morbidity (McFarlane, 2010). There has been a plethora of studies examining the association between PTSD, trauma exposure, physical illness, and somatization, and various systematic reviews have analyzed and described the extant research evidence. An early systematic review by Qureshi et al. (2009) found evidence for a consistent association between PTSD and arthritis, however, mixed results were observed for conditions such as diabetes, coronary heart disease, and stroke. In a more comprehensive systematic review of 62 studies, Pacella et al. (2013) reported a significant association between PTSD and overall poorer physical health outcomes. This encompassed general health symptoms, medical conditions, and health-related quality of life. Gupta’s (2013) review further emphasized the link between PTSD and diverse medical conditions by highlighting the severity of PTSD symptoms to be significantly associated with an increased risk of physical conditions such as hypertension and coronary heart disease. Sleep disturbances, such as sleep paralysis, were also prevalent in PTSD patients, suggesting a multifaceted impact on physical health. Afari et al.’s (2014) systematic review of 71 studies indicated that individuals with reported exposure to trauma were more likely to have functional somatic syndromes, with PTSD also identified as a contributor to cardiovascular and immune-mediated disorders. Lastly, Ryder et al.’s (2018) meta-analysis underscored a robust association between PTSD and increased risks of cardiovascular, metabolic, and musculoskeletal disorders. Collectively, these studies emphasize the intricate connection between PTSD and various physical health outcomes.

Poorer physical and somatic health status also appear to be associated with stressful life experiences such as bereavement. Parkes (1964) was among the first to show a significant correlation between bereavement and physical health in older adults by reporting a 65% increase in medical consultation rates among a sample of widows following bereavement. Large-sample cross-sectional research from Thimm et al. (2020) also demonstrated that severe grief reactions in elderly individuals were significantly associated with self-reported physical health problems as well as an increased use of health services. Additionally, Sillis et al. (2022) and Toblin et al. (2012) have shown that this association was also present in samples of younger people by reporting significant associations between grief and somatic complaints among bereaved university students and infantry soldiers. Moreover, a systematic review by Ennis and Majid (2021) found a significant, positive relationship between bereavement and adverse physical and physiological health outcomes, including inflammation, cardiovascular risk, chronic pain, and mortality.

A significant issue in the field of bereavement has been the lack of acknowledgment of enduring, distressing grief reactions as specific conditions related to grief. There has been a warranted reluctance to pathologize any form of grief, leading to inconsistencies in its definitions and measurement. As a result, depression was often diagnosed instead. However, the inclusion of Prolonged Grief Disorder (PGD) in the 11th Revision of the International Classification of Diseases (ICD-11: WHO, 2019) and the Diagnostic and Statistical Manual of Mental Disorders, Fifth Edition, Text Revision (DSM-5-TR) (American Psychiatric Association, 2022) has facilitated a more standardized approach to the study of grief.

On the other hand, the published peer-reviewed literature exploring PGD and physical health problems has also not been systematically examined since PGD was officially classified as a mental health disorder. To address this, we conducted a systematic review of the scientific literature to investigate the association between ICD-11 PGD, and outcomes related to physical health and somatic complaints among bereaved individuals. By synthesizing existing research, this review aims to provide a clearer understanding of the impact of PGD on physical and somatic health, which could inform clinical practices, guide future research, and ultimately contribute to improved care for bereaved individuals. This review represents the first comprehensive assessment of the evidence for associations between ICD-11 PGD, and physical and somatic health outcomes since PGD’s inclusion in the ICD-11.

## Method

The protocol for this systematic review was preregistered at the PROSPERO repository (CRD42023471080) on 10/10/2023 (for access, see Cunningham et al., 2023S). To ensure transparency and completeness in the processing and reporting of the results, the PRISMA 2020 guidelines (Page et al., 2021) were adhered to.

### Inclusion and Exclusion Criteria

This systematic review incorporated any form of quantitative studies that met the following inclusion criteria:

1. The study reported original, empirical research published in peer-reviewed journals, that utilized quantitative and validated measures of Prolonged Grief Disorder (PGD) and physical or somatic illness.
2. Investigated the association between PGD symptoms from standardized assessment tools and physical and somatic health symptoms.
3. Included a report of quantitative measures of association or group difference such as correlations, odds ratio, *t*-test, etc.

The exclusion criteria were:

1. Non-peer reviewed published research studies.
2. Research that did not employ a quantitative methodology.
3. Single-item quantitative scale measurement of PGD or physical or somatic illness.
4. Non-English language.
5. Studies prior to 2018.

### Search Strategy

Four electronic databases Web of Science, MEDLINE, Cochrane Library, and PsycINFO up to the 10^th^ of October 2023 were searched using full-text terms to identify studies reporting an association between PGD, and physical and somatic health symptoms. The search was limited to research studies published in the English language since 2018 that underwent peer review. Searches were conducted using Boolean operators of the following search terms:

“prolonged grief disorder” OR “prolonged grief” OR “traumatic grief” AND “somatic symptoms” OR “physical illness”.

In addition, reference lists of selected studies were screened for any other relevant study.

### Reporting Guidelines

This article was prepared in accordance with the PRISMA (Preferred Reporting Items for Systematic Reviews and Meta-Analyses) guidelines (Page et al., 2021). Adherence to PRISMA standards ensures that the research was reported with transparency and rigor, providing a clear, comprehensive, and reproducible account of the systematic review process. Following these guidelines enhanced the quality and integrity of our research findings.

### Data Collection, Extraction and Quality Assessment

After identifying studies that met the inclusion/exclusion criteria, the researchers retrieved the full-text articles. Two independent reviewers (J.C and C.C) assessed the articles for eligibility, and any disagreements were resolved by consensus. The reviewers were not blinded to the journals or authors of the studies. The researchers created a standardized data extraction sheet to gather information on publication details, study location, methodological features (such as sample size and study design), exposure and outcome measures, PGD type, and the scales used for physical and somatic health outcomes (Supplementary Table 3). The evaluation then focused on the appropriateness of quality assessment tools to measure the level of bias in each study. The resultant tool was a modification of the two most relevant instruments. The Joanna Briggs Institute critical appraisal checklist for analytical cross-sectional studies (JBI) (Joanna Briggs Institute, 2017a) (Supplementary Table 1) was applied to cross-sectional studies, while the JBI critical appraisal checklist for cohort studies (Joanna Briggs Institute, 2017b) (Supplementary Table 2) was employed for longitudinal studies. The description of effect sizes were based on Cohen (1988) descriptions of mean difference (small *d* = 0.20, medium *d* = 0.50, and large *d* ≥ 0.80) and correlations (small *r* = .10, medium *r* = 0.30, and large *r* ≥ .50).

## Results

**Figure 1:**
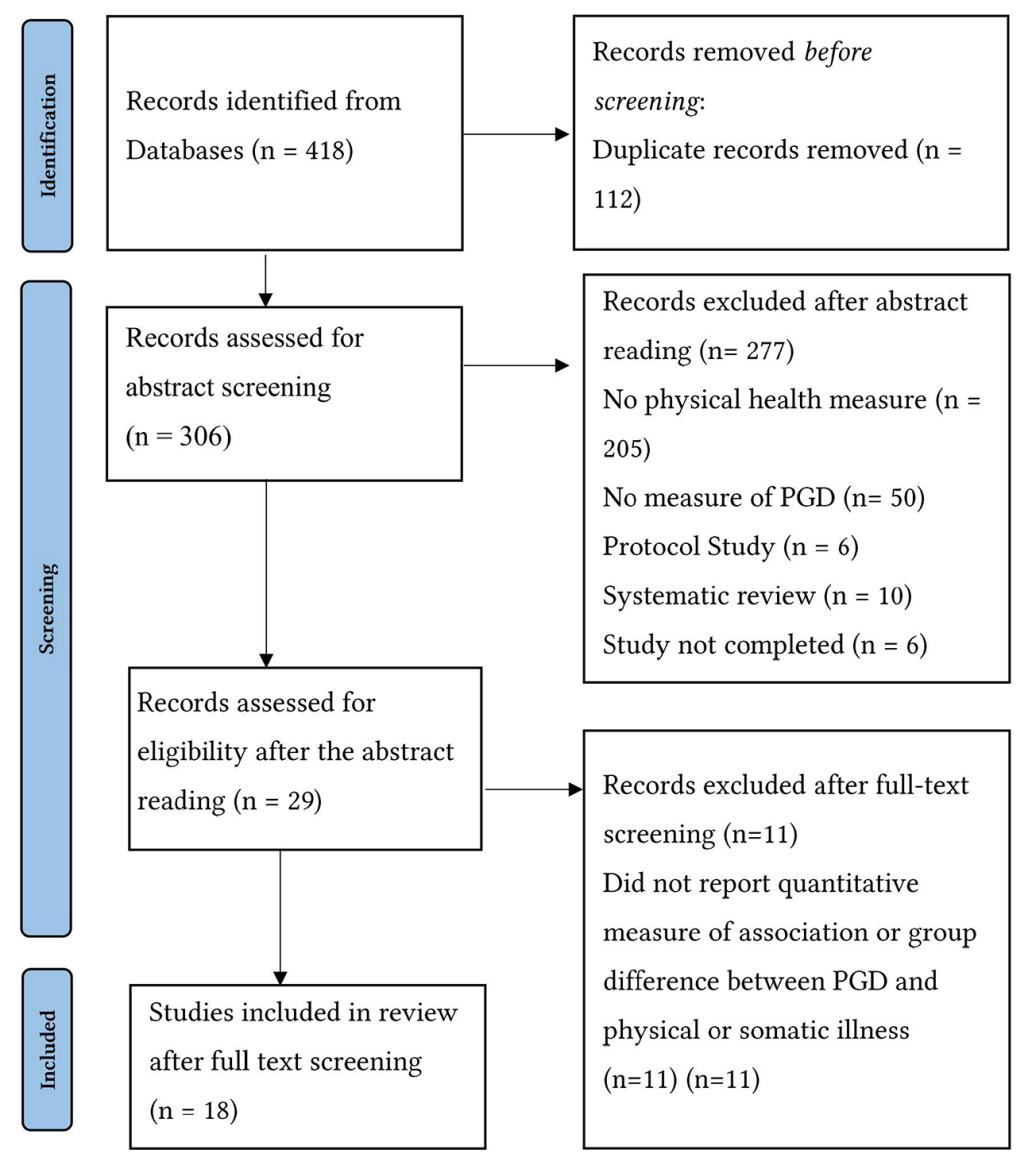
PRISMA Flow Diagram Showing the Process for Search and Selection of Studies

Details of the search and selection of studies is presented in Figure 1. Out of the initial screening based on title and abstract, 418 articles were identified, 112 of which were duplicates, and once removed, 306 articles remained. There was a high degree of agreement between the two reviewers (24 and 25 articles) in selecting articles that met the inclusion criteria (kappa = .62, *t* = 10.90, *p* < .001). After full-text screening and discussion, a final set of 18 articles were selected to take forward to full review.

Results of the reviewed studies are summarized in Table 1, covering information on associations between PGD, and physical and somatic health outcomes, mode of bereavement, sample characteristics, study design, measures, main findings, and risk of bias.

**Table 1:** Summary of Associations Between PGD and Physical, Somatic Health Outcomes

Study	Mode of bereavement (natural, sudden/unexpected/ specific illness) Time since bereavement	Sample Size and Characteristics	Study Design (Cross-sectional/longitudinal) Single group or case-control/comparison	Measures of PGD and Physical and Somatic Health	Main findings	Risk of Bias
Lundorff et al. (2020) Denmark	Loss of a spouse (Natural) 2, 6, and 11 months post-loss	*N* = 857 Female: 69.8% Male: 30.2% Mean age: 70.30	Prospective Longitudinal Single group 11-months post loss	The 13-item Prolonged Grief-scale (PG-13; Prigerson et al., 2009); Revised ICG-R (Prigerson & Jacobs, 2001); The Short-Form Health Survey (Ware et al., 1996)	Physical health significantly predicted the moderate-stable class, EST = -0.041, *SE* = 0.016, *p* = .008 which also included substantial proportions of probable PGD cases, and approached significantly as a predictor of the prolonged grief class EST = -0.041, *SE* = 0.016 *p* = .052	Low
Killikelly et al. (2020) Cross-National study China, Switzerland and the United States	Loss of a loved one (Natural) 6 to 36 months	*N* = 539 Chinese Speaking: 325 German speaking: 214 Female: 72.4% Male: 27.6% Mean age total: 35.39 Chinese sample: 33.14 German sample: 38.71	Cross-sectional Comparison group	International ICD-11 Prolonged Grief Disorder Scale (Killikelly & Maercker, 2017) The Somatic Symptom scale (Gierk et al., 2014)	Correlation coefficients between PGD (IPGDS) and somatic symptoms (SSS-8) showed moderate-level relationships for each of the three IPGDS scales for both samples. Chinese Speaking sample: IPGDS 32 items & SSS-8 = .538, IPGDS 13 items & SSS-8 = .480, and IPGDS standard with cultural supplement & SSS-8 = .540. German Speaking sample: IPGDS 32 items & SSS-8 = .508, IPGDS 13 items & SSS-8 = .458 and IPGDS standard with cultural supplement & SSS-8 = .514	Low
Vogel et al. (2021) Germany	Loss of a loved one Natural at least 6 months previously	*N* = 20 Female: 80% Male: 20% Mean age: 56	Prospective Longitudinal Single group 3 month	PG-13 (Prigerson et al., 2009) The Screening for Somatoform Disorders (SOMS-7D; Rief & Hiller, 2003)	There were no significant differences between the PGD group before and after the person-centered therapy intervention in regard to somatoform symptoms (SOMS-7D) t0-t1 *d* = 0.07, t0-t2 *d* = 0.29 *p* = .665	Moderate
Miller et al. (2020) United States	Loss of a loved one Illness: Cancer 6 to 15 months post-loss	*N* = 198 Female: 61% Male: 39% Mean age: 64.40	Prospective Longitudinal Single group study: However latent class mixture modeling is used to characterize caregiver health by identifying distinct profiles 15 months post loss	PG-13 (Prigerson et al., 2009) Overall health was assessed with 3 separate measures: a single self-report item, The health subscale of Caregiver Reaction Assessment (Given et al., 1992), and The Meeting Physical Demands subscale of the Perceived Self-Care and Daily Living Competencies Scale (Caserta et al., 2004; Utz et al., 2012).	Two distinct health profiles were identified in the total sample. Poorer Health profile group (*n* = 49; 25%) had significantly greater health impact from caregiving *d* = 0.85 (*p* < .0001), more self-reported health problems *d* = 0.53 (*p* = .002), and greater difficulty meeting the physical demands of daily life *d* = 1.16 (*p* < .0001) than the distinct profile (*n* = 149). Regression models showed that having a poorer caregiver health profile was a significant predictor of higher levels of grief symptoms *d* = 4.62 (*p* < .001) in the subsample of participants who were eligible for the bereavement analyses (*N* = 81).	Moderate
Marcussen et al. (2021) Cross-National study Denmark, Australia and Norway	Loss of a parent Cancer, sudden unexpected, suicide and chronic disease does not provide data on the time since bereavement	*N* =190 Female: 91% Male: 9% Mean age: 17.90	Cross-sectional Comparison group	The PG-13 (Prigerson et al., 2009) The CMDQ 36 (Lu et al., 2008; Tebeka et al., 2016). (Bodily distress syndrome subscale)	Prolonged grief and bodily distress syndrome showed a weak correlation at .24. There was a significant difference between the divorced parental death group *n* = 52 compared to the non-divorced parental death group *n* = 130 on bodily distress syndrome *d* = 0.375 *p* = .04. The risk of bodily distress syndrome was found to be significantly associated with parental divorce before parental death B = 3.53, *p* = .009.	Moderate
Lenger et al. (2020) Denmark	Loss of a patient caregivers who experienced the death of patients 6 months after bereavement	*N* = 2,125 Female: 70% Male: 30% Mean age: 62.00	Prospective Longitudinal Single-group However, sample split into with PGD and without PGD 6 months post-loss	The PG-13 (Prigerson et al., 2009) Short Form Health Survey-36 (SF-36) (Ware, 1999) Subscales: Physical functioning, role-physical bodily pain, and general health	Poor physical health status during caregiving predicted prolonged grief disorder: odds ratio 1.05 (95% CI [1.04, 1.07]). The physical subscales of physical functioning odds ratio 1.02, (95% CI [1.02, 1.03]). Role physical odds ratio, 1.02, (95% CI [1.01, 1.02]). Bodily pain odds ratio 1.03, (95% CI [1.02, 1.03]). and general health odds ratio 1.04, (95% CI [1.03, 1.04]). all predicted prolonged grief disorder.	Moderate
Zhou et al. (2020) China	Loss of an only child Violent and non-violent 6 months after bereavement	*N* = 1,030 Female: 62% Male: 38% Mean age: 59.91	Cross-sectional Single-group	The PG-13 (Prigerson et al., 2009) The presence of chronic physical diseases was assessed through a series of binary questions. (Yin et al., 2018). Cumulative Illness Rating Scale (Linn et al., 1968) The number of chronic physical diseases was calculated and coded into a score ranging from zero to six.	More comorbid chronic physical diseases were significantly related to the increased risk of Prolonged grief disorder *t* = 10.25, β = .33 (95% CI [1.03, 1.51])	Low
Zhang et al. (2020) China	Loss of an only child Disease and accident meantime post-loss 7.6 years	*N* = 149 Female: 60% Male: 40% Mean age: 62.25	Cross-sectional Comparison study	The PG-13 (Prigerson et al., 2009) Information about whether the participants had underlying chronic diseases was recorded. Number of outpatient visits for physical health or other reasons in the past year. It was ranked in 4 levels.	The overall morbidity of osteoarthrosis in the PGD-positive group was significantly higher than that in the PGD-negative group (χ2 = 7.18, *p* < .007). There was no significant difference in the number of hospital visits between the two groups.	Low
Pohlkamp et al. (2019) Sweden	Loss of a child Cancer 1 to 5 years after loss	*N* = 225 Female: 59% Male: 41% Mean age: 46.00	Cross-sectional Single-group study	The PG-13 (Prigerson et al., 2009) The Insomnia Severity Index (Morin, 1993)	In symptoms of insomnia, there was no significant effect of years since loss, F 4 = 1.12, .35 and no difference between genders, F 1 = 1.92, .17. There was no significant interaction between years since loss and gender on insomnia F 4 = 1.16, .33.	Moderate
Sveen et al. (2020) Sweden	Loss of a significant other Traumatic event, in the past 5 years	*N* = 123 Female: 81% Male: 19% Mean age: 37.85	Prospective Longitudinal Subsamples: comparison group Ongoing longitudinal study (TRACES study)	The PG-13 (Prigerson et al., 2009) The Symptom Checklist 27 (SCL-27) (Hardt et al., 2004)	PG-13 correlations with the somatization subscale were stronger in the bereavement group 0.57 (*p* < .001) compared to the comparison group 0.29: *Z* value = 1.77. There were no significant differences between the bereavement group *n* = 72 and the comparison group *n* = 51 on Somatization, D = -0.145.	Moderate
de Lang et al. (2023) Netherlands	Loss of a loved one, Natural accident and suicide 1 month to more than 5 years	*N* = 343 Female: 88% Male: 12% Mean age: 54.00	Prospective Longitudinal Single-group 1 year post-loss	Traumatic Grief Inventory Self-Report Plus (TGI-SR; Lenferink et al., 2022). Based on the TGI-SR (Boelen & Smid, 2017) The Insomnia Severity Index (Morin, 1993)	Correlations between prolonged grief and insomnia symptoms all showed a moderate relationship between the two variables across the three time points at 6 month intervals. PGS at time 1 displayed a weaker correlation over time against insomnia symptoms over time (T1 .39), (T2 .37) (T3 .35) PGS at time 2 ebbed and flowed as a correlation over time against insomnia symptoms overtime (T1 .35), (T2 .47) (T3 .42) PGS at time 3 displayed a stronger correlation over time against insomnia symptoms over time (T1 .36), (T2 .44) (T3 .49). All correlations are significant, *p* < .001 Participants with higher traits of prolonged grief symptoms also reported higher traits of insomnia symptoms *b* = .022 (*p* < .001). For insomnia symptoms, there was a significant autoregressive path (*p* = .011) and a cross-lagged effect from insomnia to prolonged grief symptoms *b* = .023 (*p* = .028).	Moderate
Hennemann et al. (2023) Cross-National study Germany, Switzerland and Ireland	Loss of a close loved one natural, accident, suicide, substance abuse, homicide, and natural disaster. No specific duration since the loss	*N* = 1,337 Female: 76% Male: 24% Mean age: 23.74	Cross-sectional Single-group	International ICD-11 Prolonged Grief Disorder Scale (Killikelly & Maercker, 2017) Somatic Symptom Scale (Gierk et al., 2014)	The direct effect of PGD on somatic symptom distress remained significant when including mediators (c = 0.03, *p* = .003), indicating a partial mediation of somatic symptom distress. 23% of the variance in explaining somatic symptom distress was explained by prolonged grief disorder *b* = 0.48 *p* = < .001. Two-thirds of individuals with possible PGD reported high or very high levels of somatic symptom distress in the SSS-8, which is remarkably higher than prevalences in the general population Non-PGD *M* = 6.88 PGD *M* = 12.91 *p* < .001, *d* = 0.90.	Low
Carlsson et al. (2023) Sweden	Loss of family member cardiac arrest six months after loss	*N* = 108 Female: 69% Male: 31% Mean age: 61.50	Cross-sectional Single-group: However, subsamples of Spouses and non-spouses were conducted	11 items of the 13-item PG-13 (Prigerson et al., 2009) The RAND-36 measured health-related quality of life (Hays & Morales, 2001)	Spouses reported more problems with symptoms of prolonged grief and self-reported health than non-spouses (*p* < .001). No significant differences were found between spouses and non-spouses in terms of symptoms of prolonged grief and self-reported health. 25% of family members in the present study reported their general health as fair or poor and their health to be worse compared to a year ago	High
Palitsky et al. (2023) United States	Loss of close relative Natural within the past year	*N* = 59 Female: 69% Male: 31% Mean age: 66	Cross-sectional Single-group	The PG-13 (Prigerson et al., 2009) GE Dinamap Pro 100 BP Monitors: Provided measures of SBP Systolic blood pressure and Diastolic blood pressure DBP	Increases were observed in SBP from baseline (mean [standard error], or *M* [*SE*] = 124.32 [15.01] mm Hg) to immediately post-GR (mean [standard deviation], *M* [*SD*] = 145.43 [25.17], *p* < .001, 95% CI [16.68, 25.52]). DBP also increased from baseline (*M* [*SD*] = 69.05 [8.47]) to immediately post-GR (*M* [*SD*] = 77.15 [10.67], *p* < .001, 95% CI [5.87, 10.34]). Prolonged grief disorder also significantly predicted SBP (B = 0.447, *SE* = 0.215, *p* = .042, 95% CI [0.024, 0.871]).	Low
Kaiser et al. (2022) Germany	Loss of a loved one hematological cancer time since loss not outlined	*N* = 87 Female: 83% Male: 17% Mean age: 47.32 Intervention G = 47.80 WCG = 46.84	Prospective Longitudinal A randomized controlled trial with a waitlist control group 1 year post-loss	The German version of the ICG (Prigerson et al., 1995) 12-item Short-Form Health Survey (Bullinger, 1995)	No significant group interaction was found for prolonged grief and physical health, sleep quality, or somatization. A significant within-group effect of time was found in the IG and the WCG for prolonged grief and somatization at *p* = .03 (WCG) and *p* < .001 (IG) *D* = -0.01	Low
Comtesse et al. (2020) Germany	Loss of a child, partner, parent and other. natural and un-natural 6 months post-loss	*N* = 113 Female: 81% Male: 19% Mean age: 51.68	Prospective Longitudinal Comparison group	The PG-13 (Prigerson et al., 2009) The Screening for Somatoform Disorders (SOMS7D; Rief & Hiller, 2003)	There was no significant difference between the PGD/PGD emp groups and non-PGD/ PGD emp groups in somatization *t* = 2.16 D = 0.643 and *t* = 1.62 D = 0.39. The persistent complex bereavement disorder group showed an approaching significance difference result *p* = .055 compared to the non-PCBD group D = 0.136	High
Maccallum and Bryant (2020) Australia	Loss of a partner, child, parent Sibling or other medical, accident, suicide and Homicide, 6 months after loss	*N* = 215 Female: 82% Male: 18% Mean age: 49.24	Cross-sectional Single-group	The PG-13 (Prigerson et al., 2009) The WHOQOL-BRIEF (Power et al., 1999)	Regularized partial correlation network analysis showed a significant negative association between prolonged grief disorder and physical health EL = -0.02	Moderate
Yıldırım (2023) Turkey	Loss of first-degree relative COVID-19, natural and unnatural deaths 12-24 months after loss	*N* = 68 Female: 85% Male: 15% Mean age: 45.35 PGD group = 41.90 Non PGD group = 48.80	Cross-sectional Single-group: however, subgroups of PGD and NO PGD were used	The PG-13 (Prigerson et al., 2009) Insomnia Severity Index (Morin, 1993)	Positive correlations between PGD severity and insomnia (*r* = 0.501; *p* < 0.01) There was a significant difference between the PGD group *n* = 30 and the Non PGD group *N* = 38 in severity of insomnia *t* = 2.63 *p* = .011	Low

Across the 18 studies eligible for examination, 13 (72%) demonstrated a significantly strong or moderate association between PGD and physical or somatic illness. This was displayed across divergent research designs, types of loss and different somatic and physical health problems. Cross-sectional and longitudinal designs were used in all studies. Using a cross-sectional design Killikelly et al. (2020) reported a moderate correlation between PGD (three IPGDS sub-scales) and somatic symptoms, and this is consistent with Hennemann et al. (2023) who reported that a significant proportion of variance (*R*^2^ = 23%) in somatic related distress was attributed to PGD. In contrast, Maccallum and Bryant (2020) identified a negative association between prolonged grief and physical health. In prospective longitudinal studies, Comtesse et al. (2020) found no significant differences in somatization between individuals with PGD and those without it. In contrast, Sveen et al. (2020) highlighted stronger correlations between prolonged grief and somatization in bereavement. Vogel et al. (2021) showed no significant differences in somatoform symptoms pre and post-person-centered therapy, while Kaiser et al. (2022) found no significant group interaction but observed within-group effects over time. However, both intervention studies featured small sample sizes and a 4:1 ratio of females to males, impacting statistical power and generalizability.

The types of loss reported in the studies included in this review were mostly losing a child, spouse/partner, or parent as well as the losses of patients and family members. Zhou et al. (2020) and Zhang et al. (2020) examined Chinese parents who had lost an only child and they reported associations between an increased risk of PGD and chronic physical diseases. Studies on spousal loss by Lundorff et al. (2020) and Carlsson et al. (2023) identified spousal grief symptoms as a predictor of physical health problems. Marcussen et al. (2021) found a strong correlation between prolonged grief and bodily distress syndrome in a sample who had experienced parental loss. Similarly, Lenger et al. (2020) and Miller et al. (2020) showed a significant association between prolonged grief symptoms and poorer physical health in a sample of bereaved caregivers. These findings suggest that the type of relationship with the deceased may influence the nature and severity of health outcomes associated with prolonged grief, with different relationships potentially leading to specific patterns of physical and somatic symptoms.

There were specific health outcomes that were found to be associated with grief. Yıldırım (2023) and de Lang et al. (2023) both reported a significant association between grief severity and insomnia, although this was not replicated in the Pohlkamp et al. (2019) study. The diversity of outcome types that have been investigated is reflected in the study by Palitsky et al. (2023) who found a significant association between PGD and systolic and diastolic pressure.

Supplementary Tables 1 and 2 show the comprehensive evaluation of bias risk for each study, conducted through the Joanna Briggs Institute critical appraisal checklist for analytical cross-sectional and cohort studies. This showed that 40% of cross-sectional studies exhibited moderate to high levels of bias, in contrast to the higher rate of 75% for the longitudinal studies. Significant heterogeneity was also noted. The primary bias in cross-sectional studies stemmed from the lack of control over confounding variables. Few studies controlled for participant’s previous physical health status, which is significant since individuals experiencing loss tend to be older, and older individuals tend to have more physical health complaints (James et al., 2018; Wu et al., 2022). In contrast, incomplete follow-up in cohort studies contributed to the most common element of potential bias.

## Discussion

This is the first systematic review of peer-reviewed published studies assessing the association between ICD-11 prolonged grief disorder (PGD) and outcomes related to physical and somatic health among bereaved individuals since PGD was included in the ICD-11. Among the 18 eligible studies, 13 (72%) reported moderate (Carlsson et al., 2023; de Lang et al., 2023; Killikelly et al., 2020; Lenger et al., 2020; Lundorff et al., 2020; Sveen et al., 2020) to strong associations (Hennemann et al., 2023; Marcussen et al., 2021; Miller et al., 2020; Palitsky et al., 2023; Yıldırım, 2023; Zhang et al., 2020; Zhou et al., 2020) between PGD and physical or somatic illness. There were a number of studies that reported non-significant associations, or failed to report *p*-values and were unclear in describing effect sizes (Comtesse et al., 2020; Kaiser et al., 2022; Maccallum & Bryant, 2020; Pohlkamp et al., 2019; Vogel et al., 2021). It appears that there is reliable scientific evidence, with a relatively low risk of bias, that the experience of prolonged grief is associated with poorer physical health and a higher risk of somatization. This prompts inquiry into the underlying mechanisms connecting these phenomena. Several theoretical frameworks, including attachment theory, the stress response syndrome, and the dual-process model, offer potential explanations for these observed associations.

First, attachment theory (Bowlby, 1958, 2018) provides a conceptual basis for understanding the substantial and moderate associations observed between PGD and intimate types of loss. The loss of a child, especially for mothers, has been shown to produce higher rates of PGD compared to other close loved ones (Buur et al., 2024; Goldstein et al., 2019). Attachment theory helps explain these findings due to the intense emotional bonds between parents and children, making such losses especially devastating. Moreover, the significant associations in our review studies by (Zhang et al., 2020; Zhou et al., 2020), indicate an increased risk of PGD and chronic physical diseases among Chinese parents who had lost an only child. Attachment anxiety has also been shown to predict membership into PGD groups over depression and low-symptom groups, demonstrating incremental predictive ability for both prolonged grief and somatic symptoms (Field et al., 2005; King & Werner, 2012; Maccallum & Bryant, 2018). In light of this, future research could employ quantitative measurement scales and tools for assessing attachment styles based on attachment theory. This could explore associations between attachment types and physical and somatic health outcomes for individuals meeting ICD-11 PGD criteria. If an association between attachment styles and physical/somatic health outcomes were found to be consistent, such findings may help shape practices and policies, such as identifying profiles of attachment types that pose a high risk of physical/somatic health outcomes.

Second, Horowitz’s (1986) stress response syndrome (SRS) offers a robust framework for understanding the significant associations between PGD, insomnia, and excessive blood pressure. The SRS delineates between psychological and physiological responses that individuals may undergo following traumatic or highly stressful events. This persistent state of hypervigilance has the potential to magnify the grieving process and contribute to mental health challenges, adversely impacting somatic and physical well-being (Joiner et al., 1999; Riemann et al., 2010). Regarding insomnia, the SRS would explain heightened emotional distress during nighttime, exacerbating the challenges of coping with complicated grief in solitude (Baker et al., 2016; Germain et al., 2005, 2006; Lancel et al., 2020). Furthermore, elevated blood pressure in prolonged grief sufferers may stem from persistent emotional distress and difficulties in adapting to loss, triggering complex stress responses (Mason & Duffy, 2019). In consideration of this evidence, future research could utilize biological markers and neuroimaging techniques to study hyperarousal in PGD, insomnia, and elevated blood pressure. Objective sleep monitoring (polysomnography or actigraphy) could quantify disruptions in sleep architecture. Results may show correlations between hyperarousal markers and specific sleep parameters, supporting interventions such as cognitive-behavioral therapy for insomnia (CBT-I). Advocating for CBT-I inclusion in treatment plans and workplace policies accommodating insomnia due to prolonged grief could be significant. Identifying factors moderating prolonged grief and elevated blood pressure may also inform tailored interventions and prevention strategies.

A broader perspective on the association between PGD and physical health may also be gained by examining how chronic stress and inflammation, which elucidate similar relationships in other mental disorders such as Post-Traumatic Stress Disorder (PTSD) and Major Depressive Disorder (MDE), apply to PGD. Both PTSD and MDE are linked to prolonged activation of the stress response, leading to increased inflammation (Ehlert et al., 2001; Slavich & Irwin, 2014; Wichmann et al., 2017). This inflammatory process contributes to various physical health issues, including cardiovascular disease and metabolic disorders (Black & Garbutt, 2002; Liu et al., 2017). Given that PGD involves sustained emotional distress, analogous stress-induced inflammatory pathways may also underlie the physical health problems observed in PGD. Moreover, PTSD and MDE are associated with somatic complaints such as chronic pain and gastrointestinal issues (Gupta, 2013; Thom et al., 2019), which may similarly manifest in PGD as physical symptoms due to intense grief and emotional turmoil. By exploring these parallels, researchers may gain a deeper understanding of the mechanisms through which PGD impacts physical well-being, thus guiding future research and clinical practice.

Lastly, the dual process model of coping with bereavement (Stroebe & Schut, 1999) offers a bidirectional insight into the significant associations between PGD and physical/somatic illness following unnatural loss through loss-oriented and restoration-oriented stressors (Tur et al., 2022). Unnatural or traumatic loss poses unique challenges to the grieving process, triggering intense emotions such as shock, disbelief, and intrusive thoughts (Layne et al., 2018; Lobb et al., 2010; Walsh, 2007). These emotions fall under loss-oriented stressors, as they prompt individuals to face the reality of their abnormal loss. Simultaneously, coping with the aftermath of unnatural loss involves practical challenges, such as legal processes, funeral arrangements, and dealing with the societal aftermath. It is highly plausible that individuals experiencing unnatural loss may oscillate between addressing their emotional pain and engaging in such constructive tasks. For instance, someone grieving the sudden abnormal loss of a loved one in an accident may alternate between processing the emotional trauma and dealing with the administrative aspects, such as legal procedures or insurance matters. This consistent fluctuation may create cognitive dissonance (Festinger, 1957) in those experiencing unnatural loss which may elucidate the substantially significant associations observed between PGD and physical and somatic illness through abnormal loss circumstances. For example, Dickerson and Kemeny (2004) have shown how stressors that involve social-evaluative threats a key component of cognitive dissonance lead to significant increases in cortisol levels, which has been shown to suppress the immune system, making individuals more vulnerable to illness. In the context of PGD, the ongoing internal conflict and chronic stress may result in a sustained physiological response, thereby weakening immunity and increasing susceptibility to physical ailments. Additionally, cognitive dissonance has also been linked to an increased risk of cardiovascular disease. Linden et al. (2007) found that stress arising from conflicting emotions or behaviours which are key elements of cognitive dissonance significantly heightens the risk of hypertension and other cardiovascular problems. For individuals with PGD, the persistent cognitive dissonance they experience may intensify their stress, thereby increasing the likelihood of developing cardiovascular issues. Keeping this in consideration, future research could refine and adapt existing prolonged grief and coping scales to better align with the nuances of the dual process model. A dual-process model questionnaire could focus on addressing specific components of prolonged grief that contribute to cognitive dissonance and potential physical and somatic health complications. Policymakers could integrate such screening tools into routine health assessments, while employers and community organizations could offer more targeted support programs.

The studies under review exhibited both strengths and limitations. They notably demonstrated consistency in measuring PGD, alongside showcasing geographical and cultural diversity, which enriched external and ecological validity. However, this cultural diversity may explain the assorted findings found across the reviewed studies regarding the strength of the association between PGD and physical and somatic health outcomes. Future research could investigate this by examining how cultural factors influence this relationship, potentially through incorporating culturally sensitive measures in assessments. The majority of studies also presented substantial sample sizes, often supported by reported power analyses. On the other hand, the bias risk evaluation revealed differences in bias levels. Cross-sectional studies tended to have lower bias than longitudinal studies. Moderate bias was noted in cross-sectional studies, while higher bias was observed in cohort studies. Both designs exhibited relatively low levels of high bias. Future studies, especially in cohort designs, can benefit from proactive strategies and experimental designs to mitigate bias and enhance generalizability. However, given the intrinsic difficulty in manipulating grief as an emotional state in experimental settings, researchers must approach this challenge with caution and creativity. Methodologically, it’s noteworthy that the majority of studies relied on self-reported measures that lacked control for confounding variables, while only 44% utilized longitudinal methodology, potentially impacting internal validity and result interpretability. Substantial heterogeneity was observed among the studies analyzed, with four distinct scales employed to evaluate physical health and five to measure somatic health outcomes. Moreover, three studies adopted alternative quantification methods, including the use of monitors, chronic disease assessments, and outpatient visits. This disparity in measurement complicates direct result comparisons, as it’s unclear if differences stem from variable characteristics or scale usage. Developing universal physical and somatic health scales could address this, offering standardized measures across cultures. This would aid cross-cultural comparisons and deepen our understanding of physical and somatic health outcomes. The included studies also exhibited a fairly high mean age of 50 which may not capture the unique prolonged grief experiences of younger individuals who may have different coping mechanisms, support structures, and life contexts compared to older adults.

In conclusion, this pioneering review on PGD’s association with physical and somatic illness exhibited numerous strengths such as the consistent measurement of PGD, substantial sample sizes, and a high level of regional diversity. However, limitations included disparities in bias levels between transverse and cohort studies, heterogeneity in attaining the measurement of physical and somatic illness and the use of self-reported measures that lacked control for confounding variables. The reviewed results revealed a hierarchy of associations. Most studies demonstrated a significantly strong or moderate association between PGD and physical or somatic illness. Notable findings include PGD’s impact on caregiver health decline, somatic symptom distress, insomnia severity, and comorbid chronic diseases such as osteoarthrosis and elevated blood pressure. These results are consistent with PTSD findings and highlight the clinically relevant effect sizes both psychologically and medically. These findings may assist in the differential diagnosis of PGD by emphasizing the unique combination of psychological and physiological symptoms, which can help distinguish PGD from other disorders such as PTSD. Given the significant impact of PGD on physical health, it is important to consider these physiological symptoms more prominently in the diagnostic process to ensure comprehensive assessment and appropriate treatment.

An important additional consideration is the impact of behavioural changes associated with PGD on overall health. PGD has been shown to cause behavioural changes that contribute to poor physical and mental health. For instance, individuals with complicated grief may engage in behaviours such as binge drinking, smoking, and a lack of physical activity (Stroebe et al., 2007). These behaviours can exacerbate chronic illness, which in turn impacts an individual's mental health and affects their ability to participate effectively in therapy (Lando, 2006). Understanding these interactions is crucial, as social withdrawal known as a common response in PGD (Szuhany et al., 2021) can lead to further physical and mental health problems. A comprehensive approach to PGD treatment must consider these behavioural changes and their impact on overall health to enhance therapeutic outcomes and support holistic recovery.

Future research avenues include integrating quantitative tools based on attachment theory for intimate losses in routine PGD screenings or employing biological markers and neuroimaging techniques to study hyperarousal in PGD, insomnia, and elevated blood pressure. Additionally, the dual process model of coping with bereavement could be utilized through a standardized questionnaire tailored to measure the framework, potentially predicting physical or somatic health issues among prolonged grief sufferers. However, future studies must prioritize methodological rigor, diverse participant samples, and ethical standards to ensure valid and applicable findings in clinical practice.

## Supplementary Materials

The Supplementary Materials contain the following items:

- **Preregistered PROSPERO Protocol** (Cunningham et al., 2023S)
- **Online Appendices** (Cunningham et al., 2025S):
  - **Appendix A**: JBI Critical Appraisal Checklist for Analytical Cross-Sectional Studies.
  - **Appendix B**: JBI Critical Appraisal Checklist for Cohort Studies.
  - **Appendix C:** Standardized Data Extraction Sheet.
  - **Appendix D:** Descriptions of the included studies.
  - **Appendix E:** References from Systematic Review.

CunninghamJ.
ShevlinM.
CerdaC.
McElroyE.
 (2023S). ICD-11 prolonged grief disorder, physical health and somatic problems: A systematic review
[Preregistration]. PsychOpen. https://www.crd.york.ac.uk/prospero/display_record.php?RecordID=471080
10.32872/cpe.14351PMC1196056740177338

CunninghamJ.
ShevlinM.
CerdaC.
McElroyE.
 (2025S). Supplementary materials to "ICD-11 prolonged grief disorder, physical health, and somatic problems: A systematic review"
[Online appendices]. PsychOpen. 10.23668/psycharchives.16037
PMC1196056740177338

## Data Availability

All materials are freely available from the corresponding author on request.

## Social Media Accounts

@mark_shevlin_

## Preregistration

The protocol for this systematic review was preregistered at the PROSPERO repository **(**CRD42023471080**),** and is available at https://www.crd.york.ac.uk/prospero/display_record.php?RecordID=471080

## Reporting Guidelines

This article was prepared following the PRISMA guidelines (Preferred Reporting Items for Systematic Reviews and Meta-Analyses) (Page et al., 2021).
